# Development of a checklist framework for kidney transplantation

**DOI:** 10.3389/frtra.2024.1412391

**Published:** 2024-06-25

**Authors:** Ramona Nicolau-Raducu, Gaetano Ciancio, Yehuda Raveh

**Affiliations:** ^1^ Departmet of Anesthesiology, Solid Organ Transplant & Vascular Anesthesia, University of Miami Leonard M. Miller School of Medicine, Miami, FL, United States; ^2^ Department of Surgery and Urology, Miami Transplant Institute/Jackson Memorial Hospital, University of Miami Leonard M. Miller School of Medicine, Miami, FL, United States

**Keywords:** checklist, kidney transplantation, perioperative, anesthesia, surgery

## Abstract

**Background:**

Kidney transplantation is the therapy of choice for end-stage kidney disease, and a fast-growing transplant procedure worldwide. Diverse clinical practices for recipients and donors' selection and management between transplant centers hinder the creation and dissemination of an anesthesia-surgical checklist.

**Methods:**

Components of the anesthesia-surgical checklist were selected after a review of the English literature using PubMed search for donor, recipient and graft protocols and outcomes of existing practices in the field of kidney transplantation. Key elements of the most relevant articles were combined with our own center's experience and formulated into the proposed checklist. The checklist is intended to be used perioperatively, once patient receives an offer.

**Results:**

The perioperative checklist centers primarily on the following donor and recipient's factors: (i) Review of the pretransplant candidate workup; (ii) Assessment of donor/graft status; (iii) Hypothermic machine perfusion parameters; (iv) Operating room management; (v) Sign out. The proposed kidney transplant checklist was designed to ensure consistency and completeness of diverse tasks and facilitates team communication and coordination.

**Conclusion:**

We present a novel standardized combined anesthesia-surgical checklist framework for kidney transplant aimed at increasing perioperative safety and streamline the perioperative care of recipients. Future validation studies will determine its clinical feasibility and post-implementation efficacy.

## Background

Kidney transplantation (KT) is the therapy of choice for patients with end-stage kidney disease, and a fast-growing transplant procedure worldwide. The World Health Organization launched The World Alliance for Patient Safety in 2004, and in 2008 the second Global Patient Safety Challenge was formulated and aims to improve surgical safety. The compilation of a procedure checklist for use in the operating room is one of the four tenets of the initiative (1). Proven benefits of surgical checklists include enhanced team communication, procedural consistency, and ultimately significant reduction in related medical errors and surgical complications (2). Within the scope of urology checklists were successfully implemented in transurethral resection of a bladder tumor, robotic nephrectomy, and radical nephrectomy with tumor thrombectomy (3–5).

To expand the ever-limited donors pool, high-volume transplant centers are generally more inclined to consider expanded criteria donors who are older (≥75 years), hypertensive, or stroke-stricken (6). However, the increased comorbidities of aging donors or donation after circulatory death are associated with poorer graft and recipient's outcomes (7, 8). Perioperative fluid and hemodynamic management determine renal allograft function and, consequently, perioperative adverse cardiac events of recipients (9). As such, it is increasingly important to optimize modifiable risk-factors and improve short- and long-term outcomes in KT recipients. In addition to the surgical technique itself, optimal outcomes of KT also require careful matching of the donor and recipient, adequate machine perfusion of graft, immunosuppression, and a set of perioperative hemodynamic and fluid goals. Notwithstanding its complexity, a universal checklist for KT is strikingly lacking. Presumably, diverse clinical practices for recipients and donors’ selection and management between transplant centers hinder the creation and dissemination of such a checklist.

This study aims to fill this gap. We propose a combined anesthetic-surgical perioperative checklist framework aiming at improving the perioperative care of patients undergoing KT.

## Method

Components of the anesthesia-surgical checklist were selected after a review of the English literature using PubMed search for donor, recipient and graft protocols and outcomes of existing practices in the field of KT. Key elements of most relevant articles were combined with our own center's experience and formulated into the proposed checklist, see Table 1. The KT checklist is intended to be used perioperatively once the patient receives an offer.

**Table 1:** Checklist for the performance of kidney transplantation.

Day of surgery/preoperative workup		
Reviewed history/comorbidities and physical exam performed	Yes	No
Redo transplantation	Yes	No
Reviewed radiological studies	Yes	No
Reviewed cardiovascular workup	Yes	No
Reviewed medication list	Yes	No
Anticoagulation Aspirin/Antiplatelets/Coumadin/others	Yes	No
Last dose <24 h	Yes	No
Reviews labs and abnormalities addressed	Yes	No
Last Potassium <5.5 mEq/L	Yes	No
Human chorionic gonadotropin (HCG) positive	Yes	No
Renal replacement therapy (Hemodialysis/Peritoneal dialysis)	Yes	No
Time on dialysis ≥5 years	Yes	No
Dialysis issues: hypotension/Midodrine usage	Yes	No
Last dialysis ≥48 h	Yes	No
Dialysis Access Arterio-Venous fistula/Dialysis catheter	Yes	No
History of central venous access thrombosis	Yes	No
Making urine >100 ml/day	Yes	No
Urinalysis: presence of proteinuria	Yes	No
Review PRA and crossmatch results	Yes	No
Graft assessment: DBD/DCD/living donor		
Reviewed donor comorbidities/KDPI	Yes	No
Reviewed donor Serology Form	Yes	No
Reviewed donor biopsy results	Yes	No
Requires renal vascular reconstruction/use of extra vessels from a deceased donor	Yes	No
Review Perfusion Pump Parameters (HMP)		
Cold Storage Time^ ^+ Total Pump Time ≤40 h	Yes	No
Pump resistance <0.3 mmHg/ml/min	Yes	No
Pump flow ≥120 ml/min	Yes	No
Systolic pressure average 30 ± 5 mmHg	Yes	No
Operating room		
Confirm patient identity, procedure, surgical site marking, team members	Yes	No
ALL required informed consent obtained	Yes	No
Type and screen/crossmatched blood	Yes	No
Reviewed allergies including rabbit exposure	Yes	No
Reviewed airway exam and NPO status	Yes	No
UNOS ABO verification by 2 staff done before anesthesia induction	Yes	No
Baseline mean BP <80 mmHg	Yes	No
Vascular access on opposite site of AV fistula	Yes	No
Peripheral intravenous access ×2	Yes	No
Arterial line	Yes	No
Need for central line/others	Yes	No
Thromboelastographic test needed	Yes	No
Antibiotics given <1 h prior surgical incision	Yes	No
Pre-medication for anti-rejection medication given	Yes	No
Anti-rejection induction medications given	Yes	No
Diuretics Lasix/mannitol given	Yes	No
Mean arterial pressure post reperfusion >100 mmHg	Yes	No
Usage of phenylephrine/ephedrine during transplant	Yes	No
Balanced crystalloid/colloid goal achieved	Yes	No
Timing documentation: kidney out of ice; total pump time; end pump parameters; reperfusion; total cold ischemia time; warm ischemia time	Yes	No
Urine production	Yes	No
Surgical drainage	Yes	No
Ureteral stent	Yes	No
Foley catheter 3 ways or 2 ways	Yes	No
Planned extubation	Yes	No
Case closure: instrument, sharp, and towel counts	Yes	No
Disposition to ICU/recovery team notified	Yes	No
Sign-out		
Pain management under control	Yes	No
Sign-out completed in PACU/ICU	Yes	No

PRA, panel reactive antibody; KDPI, kidney donor profile index; DBD, donation after brain death; DCD, donation after cardiac death; HMP, hypothermic machine perfusion; UNOS, United Network for Organ Sharing; PACU, post aesthesia care unit; BP, blood pressure; AV, arterio-venous; ICU, intensive care unit.

The perioperative checklist centers primarily on the following donor and recipient's factors:

(i) Review of the pretransplant candidate workup
(ii) Assessment of donor/graft status
(iii) Hypothermic machine perfusion parameters
(iv) Operating room management
(v) Sign out

### Checklist components

(i) Review of the workup of the recipient

A thorough review of recipient's history, medication, physical examination, recent laboratory radiological results is mandatory. Issues which may preclude transplantation should be sought after and addressed.

Patients with a cardiovascular or respiratory disease, limited functional status, or prior hospitalization should be identified, and attainment of patient's optimal medical status prior to KT should be ascertained. Cardiovascular complications continue to be the leading cause of mortality after KT. While KT is categorized as an intermediate risk surgery by the American College of Cardiology and the American Heart Association, is associated with a 1.1% perioperative cardiovascular mortality, compared to 0.7% mortality reported after major abdominal surgery (10). Preoperative referral for cardiology consultation should be guided by cardiac risk assessment of the KT candidate (11).

The recently updated Kidney Disease: Improving Global Outcomes guidelines (12) recommended the referral of potential kidney transplant candidates for evaluation at least 6–12 months before anticipated dialysis initiation in order to facilitate the identification and workup of living donors and to plan for possible pre-emptive transplantation. The reason is that the time spent on dialysis before transplantation appears to have a directly proportional negative effect on patient and/or graft survival (13). Symptomatic hypotension is a common complication in patients with end-stage renal disease (14). Midodrine users were more likely to have a longer duration of dialysis dependence, and higher levels of sensitization compared to non-users (15). In addition, they are at increased risks of developing delayed graft function (DGF), graft failure and death in the 1st year post transplant (15). Details regarding dialysis access, when last dialysis was done, amount of urine making are important in regard of intraoperative venous/arterial access, potassium level and the size of the bladder, respectively. Occurrence of proteinuria, (≥0.5 g/day), first post-transplant year, is significantly linked to graft/patient survival and usage of older donors (≥60 years), which appear more sensitive to proteinuric injuries (16). The first consideration in evaluating post-transplant proteinuria is whether it originates from the native kidneys or may be due to various allograft pathologies, and/or may be a side effect of immunosuppressive medications (17). Patients not on dialysis before transplantation may have proteinuria due to their native kidney disease (17).

Human leukocyte antigens (HLAs) are the primary determinants of alloimmunity (18). A crossmatch test is a test that determines the immunologic risk and can be done by mixing patient serum and donor cell (logistically challenging) or virtually, which is analyzing the results of 2 independently done physical laboratory tests—patient anti-HLA antibody and donor HLA typing (18). Exposure to nonself HLA antigens can lead to formation of anti-HLA antibodies, a process known as sensitization (19). This occurs primarily via three types of exposure: blood transfusions, pregnancy, and solid organ transplant (19). Panel reactive antibody (PRA) is a test that identifies sensitized patients and estimate their likelihood of finding a crossmatch-compatible donor (18). The calculated PRA (cPRA), mandated in the United States since 2009 to assess immune sensitization status (18). To improve transplant rates among highly sensitized patients, the Organ Procurement and Transplantation Network implemented key changes to the kidney allocation system in December 2014 by awarding points based on cPRA level (19). Desensitization involves the use of treatment regimens that decrease the preformed antibody levels directed toward the potential donor (19). Typically, the goal of therapy is to reduce the antibody level, so the flow cytometric crossmatch is negative or lower than a predetermined cutoff (19). Many different protocols used with varying success; most combine plasmapheresis, Intravenous immunoglobulin, rituximab, bortezomib, and early initiation of maintenance immunosuppression several weeks before the transplant (19).

(ii) Assessment of donor/graft status

There are different types of donor allograft kidneys: deceased vs. living donor. Deceased brain death (DBD) organs can come from standard criteria donors or from expanded criteria donors (ECD) (20), donation after cardiac death (DCD) (21), double kidney transplant (dual) (22) or donor with increased risk social behavior (23). Living donor can be direct or non-direct (altruistic) donation vs. pair donation or pair exchange (24).

The growing gap between demand and supply for kidney transplants has led to worldwide renewed interest in the use of ECD to increase the donor pool. Although most studies confirm lower allograft survival rates and, generally, worse outcomes than standard criteria donor kidneys, recipients of expanded donor criteria kidneys generally have improved survival compared with wait-listed dialysis patients (20, 25). The Kidney Donor Profile Index (KDPI) was introduced in the United States in 2014 to guide the decision making of clinicians with respect to accepting or declining a donated kidney. The KDPI is a more refined metric for assessing expected longevity of grafted kidneys, since it considers 10 donor factors, compared to ECD which only considers four (age, creatinine, history of hypertension, and cause of death). In addition, the ECD criterion is limited since it is a binary assessment of donor quality, while KDPI estimates donor quality on a continuous scale. The KDPI measures the quality of deceased kidney and assigns a percentage score. The KDPI is derived by calculating the Kidney Donor Risk Index (KDRI)—a numerical measure of the quality of deceased donor kidney that combines 10 donor factors, including clinical parameters and demographics. The KDRI is an estimate of the relative risk of post-operative kidney graft failure (in an average adult recipient) from a particular deceased donor compared to a reference donor. The ability of KDPI to capture only some but not all donor factors that are predictive to graft outcome, and the absence of relevant recipient variables as well as factors related to the transplant procedure are amongst its important limitations (26).

Donation after circulatory death kidney transplantation has been introduced also to address organ shortage. Two recent systematic review and meta-analysis showed that long-term DCD kidney transplant outcomes are like DBD despite a higher risk of primary non function, DGF and graft loss in the first year after transplantation (27, 28).

The concept of “increased risk” (previously referred to as “high risk”) donors was created to identify a population of deceased or living donors potentially at risk for recent acquisition of human immunodeficiency virus, or viral hepatitis (29). This period between infection and the development of antibodies is called the “serological window period.” Nucleic Acid Testing, which has been used with increasing frequency over the last decade, is now required by the Organ Procurement and Transplantation Network Policy (for hepatitis C virus and human immunodeficiency virus) for all increased risk donors (29). Even with the increased sensitivity offered by nucleic acid testing, this testing may not detect a human immunodeficiency virus, hepatitis B or C virus exposure that occurred within the three to five days prior to testing (29). In communicating the risk of donor-derived infection from any donor including those associated with donors bearing the behavioral factors, it is important to consider the risks to the potential recipient of not accepting that organ and continuing to wait for another offer (29). Further, recipients who receive organs from donors bearing these characteristics should be informed that they will be monitored post-transplant for infection with human immunodeficiency virus, or hepatitis B or C virus (29).

Procurement kidney biopsies are used to assess a deceased organ donor's kidney for organ damage and potential kidney function (30). Over the years, kidney biopsies have become more widely used, but their usage varies greatly across the country (30). Recently, Organ Procurement and Transplantation Network policy proposed minimum adult kidney donor criteria which require biopsy such us: urine output of less than 100 ml in 24 h; donor has received hemodialysis or other renal replacement therapy either during most recent hospital admission or in the course of donor management; history of diabetes, including a hemoglobin A1c of 6.5 or greater during donor evaluation and management; KDPI greater than 85%; donor age 60 years old or greater; donor age 50–59, and meets at least two of the following criteria: history of hypertension; manner of death: cerebrovascular accident; terminal creatinine of 1.5 mg/dl or greater (31). However, many transplant centers don't see this as a tool to decrease the number of unnecessary biopsies, thus increasing kidney utilization (31). Reasons behind are: (i) the donor selection criteria for biopsy are too wide; (ii) no standardized approach to the biopsy process (who recovers the sample, how it is processed, read and reported); (iii) donor hospitals do not have enough infrastructure to accommodate the proposed changes; (iv) no transplant center accountability, particularly if a biopsy is not required and the center performs their own after acceptance, leading to a turn down, (v) current data shows that renal biopsy does not significantly improve long term graft success/failure (31, 32).

(iii) Hypothermic machine perfusion parameters

Kidney grafts are often preserved initially in static cold storage and subsequently on hypothermic machine perfusion (MP). Cannon et al. demonstrated the decreased incidence of DGF but no difference in graft survival by MP when compared with cold storage alone (33). In current practice in the United States, it is common that MP is used only for a small part of the entire preservation period, potentially explaining the lack of any survival benefit in the analyses (34). Similar to our center's protocol, a kidney graft may be preserved by MP only after the graft is transferred to a local organ recovery agency; a kidney may also be preserved by MP only for a short period of time (e.g., 4 h) just to assess the quality of the graft by perfusion parameters such us: pump resistance and flow and average systolic pressure (34, 35). Prolonged cold storage time (particularly ≥6 h) before MP has a negative impact on DGF occurrence in DCD kidney transplantation (34). Long MP time (≥36 h) (and thus cold ischemia time ≥42 h) detrimentally affects DGF occurrence in both DCD and DBD kidney transplant recipients even when the grafts were mainly preserved by MP (34).

(iv) Operating room

The routine verification of recipient identity, all required consents, no per os status, allergies and type and screen/cross is followed by United Network for Organ Sharing (UNOS) ABO verification. When the transplant surgery must begin before the organ arrives in the operating room, a pre-transplant verification must take place: two licensed healthcare professionals (does not have to be the surgeon); must occur before induction of general anesthesia (unless patient is receiving continuous sedation prior to arriving the operating room; then prior to first incision). Verification MUST include: UNOS donor identification expected from the UNOS match run; expected organ AND laterality (if applicable); expected donor blood type from UNOS documentation; verify recipient in room is intended recipient; intended recipient's blood type from medical record; verification that donor and recipient are ABO compatible.

Thymoglobulin, an IgG fraction purified from the serum of rabbits immunized against human thymocytes, is commonly used as immunosuppressant in kidney transplantation (36). While there are rare reports of anaphylactic reaction to thymoglobulin due to rabbit protein allergy, this can be avoided if rabbit exposure is ruled out during preoperative evaluation (36).

Due to inability of the allograft to auto regulate blood pressure, both low and high blood pressure perioperatively could be detrimental. Chronic hypotension remained a major predictive factor for DGF development with lower graft survival if kidney transplant was done from >50 years old donor (37). On the other hand, early hypertension is common after renal transplantation, however early blood pressure control has the potential to influence the risk of allograft rejection and DGF (38). In general, during anesthesia, we aim to keep blood pressure at baseline ±20%. Transplant surgeons often are asking for a mean blood pressure of 100 mmHg at the time of reperfusion. A higher than traditional normal is linked to the need of the newly grafted kidney for optimal perfusion pressure (35). Treating hypotension can be challenging in kidney transplant because vasoconstrictor drugs such as: phenylephrine (35) and/or ephedrine (39) were linked with DGF. Phenylephrine was linked to delayed graft function especially in patients who had a drop of 30 mmHg or more in blood pressure 30 min after reperfusion (35). However, it's unlikely that phenylephrine-induced vasoconstriction is the culprit, since the effect of a bolus dose is brief, and the phenylephrine was administered before reperfusion in more than half of the recipients (35). Plausibly, intraoperative phenylephrine use is a surrogate of an unmeasured hemodynamic variable, e.g., postoperative allograft perfusion, or another clinical parameter that influences the outcome (35).

A recent published Consensus Statement of the Committee of Transplant Anesthesia of the American Society of Anesthesiologists, systematic review of the literature addressed the fluid management in kidney transplantation: types of fluids, quantity, and volume status assessment (9). Regarding the amount of fluids and monitoring volume status during kidney transplantation the Consensus Statement found that we have weak recommendation for instance: low quality evidence for larger volume fluid administration targeting higher central venous pressure during KT; use of central venous pressure as a guide to fluid administration is weakly supported; weak evidence of accelerated fluid administration during graft ischemia rather than constant infusion will lead to improved graft function; although not specific to fluid volume, avoidance of intraoperative hypotension with different thresholds support improved graft function; reports of stroke volume variation or esophageal Doppler to guide fluid administration are promising but limited (9). Regarding colloids and type of crystalloids the Consensus Statement also found weak recommendations to the use of albumin over crystalloids alone discourages the use of starches which were linked to worse outcomes. However, there is strong evidence that supports the use of balanced solutions over normal saline and (9, 40).

Enhanced recovery after surgery protocols is not limited to a specific surgical intervention, allowing for wider implementation, including in kidney transplantation. Enhanced recovery after surgery protocol, modified to address kidney transplantation unique issues, is feasible and renders low morbidity and reasonable readmission rates (41).

(v) Sign out

A brief operative note should be completed prior to patient transport to ensure accurate communication to teams in the post-operative recovery area or intensive care unit. At the time of patient handoff, the surgeon and anesthesia should speak directly with the receiving team to ensure continuity of care.

## Limitation

The proposed checklist may appear vague without any workup specifics or cutoff, since each transplant center has its own selection criteria.

The proposed checklist is geared towards ensured consistency and completeness of miscellaneous tasks. The checklist also facilitates team communication and coordination. This manuscript did not aim to address perioperative management; therefore, specifics of perioperative management were omitted.

## Conclusion

We present a novel standardized combined anesthesia-surgical checklist framework for kidney transplant aimed at increasing perioperative safety and streamline the perioperative care of recipients. Future validation studies will determine its clinical feasibility and post-implementation efficacy.
